# Engagement and Acceptability of Acceptance and Commitment Therapy in Daily Life in Early Psychosis: Secondary Findings From a Multicenter Randomized Controlled Trial

**DOI:** 10.2196/57109

**Published:** 2024-11-21

**Authors:** Evelyne van Aubel, Thomas Vaessen, Lotte Uyttebroek, Henrietta Steinhart, Annelie Beijer-Klippel, Tim Batink, Ruud van Winkel, Lieuwe de Haan, Mark van der Gaag, Thérèse van Amelsvoort, Machteld Marcelis, Frederike Schirmbeck, Ulrich Reininghaus, Inez Myin-Germeys

**Affiliations:** 1 Center for Contextual Psychiatry, Psychiatry Research Group Department of Neurosciences KU Leuven Leuven Belgium; 2 Department of Psychology, Health & Technology Faculty of Behavioural, Management and Social Sciences (BMS) University of Twente Enschede Netherlands; 3 Department of Psychiatry and Neuropsychology School for Mental Health and Neuroscience, Faculty of Health, Medicine and Life Sciences Maastricht University Maastricht Netherlands; 4 Department of Lifespan Psychology Faculty of Psychology Open University Heerlen Netherlands; 5 Center for Clinical Psychiatry, Psychiatry Research Group Department of Neurosciences KU Leuven Leuven Belgium; 6 Department of Psychiatry Amsterdam UMC University of Amsterdam Amsterdam Netherlands; 7 Department of Clinical Psychology VU Amsterdam Amsterdam Netherlands; 8 Parnassia Psychiatric Institute The Hague Netherlands; 9 Institute for Mental Health Care Eindhoven (GGzE) Eindhoven Netherlands; 10 Department of Public Mental Health Central Institute of Mental Health Medical Faculty Mannheim, University of Heidelberg Mannheim Germany; 11 ESRC Centre for Society and Mental Health and Centre for Epidemiology and Public Health, Health Service and Population Research Department Institute of Psychiatry, Psychology and Neuroscience King’s College London London United Kingdom

**Keywords:** acceptance and commitment therapy, ACT, first episode of psychosis, FEP, ultrahigh risk for psychosis, UHR, ecological momentary intervention, EMI, mobile health, mHealth, blended care, mobile phone

## Abstract

**Background:**

Acceptance and commitment therapy (ACT) is promising in the treatment of early psychosis. Augmenting face-to-face ACT with mobile health ecological momentary interventions may increase its treatment effects and empower clients to take treatment into their own hands.

**Objective:**

This study aimed to investigate and predict treatment engagement with and acceptability of acceptance and commitment therapy in daily life (ACT-DL), a novel ecological momentary intervention for people with an ultrahigh risk state and a first episode of psychosis.

**Methods:**

In the multicenter randomized controlled trial, 148 individuals with ultrahigh risk or first-episode psychosis aged 15-65 years were randomized to treatment as usual only (control) or to ACT-DL combined with treatment as usual (experimental), consisting of 8 face-to-face sessions augmented with an ACT-based smartphone app, delivering ACT skills and techniques in daily life. For individuals in the intervention arm, we collected data on treatment engagement with and acceptability of ACT-DL during and after the intervention. Predictors of treatment engagement and acceptability included baseline demographic, clinical, and functional outcomes.

**Results:**

Participants who received ACT-DL in addition to treatment as usual (n=71) completed a mean of 6 (SD 3) sessions, with 59% (n=42) of participants completing all sessions. App engagement data (n=58) shows that, on a weekly basis, participants used the app 13 times and were compliant with 6 of 24 (25%) notifications. Distribution plots of debriefing scores (n=46) show that 85%-96% of participants reported usefulness on all acceptability items to at least some extent (scores ≥2; 1=no usefulness) and that 91% (n=42) of participants reported perceived burden by number and length of notifications (scores ≥2; 1=no burden). Multiple linear regression models were fitted to predict treatment engagement and acceptability. Ethnic minority backgrounds predicted lower notification response compliance (B=–4.37; *P*=.01), yet higher app usefulness (B=1.25; *P*=.049). Negative (B=–0.26; *P*=.01) and affective (B=0.14; *P*=.04) symptom severity predicted lower and higher ACT training usefulness, respectively. Being female (B=–1.03; *P*=.005) predicted lower usefulness of the ACT metaphor images on the app.

**Conclusions:**

Our results corroborate good treatment engagement with and acceptability of ACT-DL in early psychosis. We provide recommendations for future intervention optimization.

**Trial Registration:**

OMON NL46439.068.13; https://onderzoekmetmensen.nl/en/trial/24803

## Introduction

Individuals with an ultrahigh risk (UHR) [1,2] state for psychosis are at increased risk of transitioning to a first episode of psychosis (FEP), with meta-analyses showing transition rates of 29% two years after presentation to mental health services, and up to 36% after 3 years [3,4]. These rates suggest that the UHR status temporally and phenomenologically precedes an FEP [5], which are now both conceptualized as the “early stages of psychosis” [1,2,5-7]. At a phenomenological level, psychotic experiences are an important source of distress in UHR individuals [8-10]. Moreover, UHR individuals who do not transition to psychosis nor remit show a reduction in functioning similar to FEP individuals [11-13]. As for the latter, while sustained periods of symptom remission are common after a FEP [14], persisting psychotic symptoms are associated with significant levels of distress [15,16], and for a majority of FEP individuals, impaired long-term functioning, reduced quality of life, social exclusion in work and relationships, and increased mortality have been reported [14,17]. These results highlight the importance of early interventions to prevent transition to more severe stages, as well as to alleviate psychotic distress and improve functioning.

Currently available psychological and pharmacological interventions can effectively reduce the transition from UHR to FEP, with cognitive behavioral therapy (CBT) showing the most robust effects [18]. However, these meta-analyses did not find significant improvements in psychotic distress, functioning, quality of life, or other affective symptoms [18,19]. One therapy that may successfully target these outcomes is acceptance and commitment therapy (ACT) [20]. ACT is a third-wave behavioral therapy that, unlike traditional CBT, focuses on changing the relationship between an individual and their thoughts and experiences, rather than reappraising them [20]. From the perspective of ACT, individuals tend to avoid, suppress, or control unwanted psychological experiences, a process known as experiential avoidance [20,21]. Previous studies showed that experiential avoidance was a mediator between the experience of daily life hassles and delusional distress [22], and that it was associated with distress related to auditory hallucinations [23], as well as with higher depression, anxiety, and stress [24] in individuals with established psychotic disorder. ACT, therefore, aims to teach individuals alternative acceptance skills to replace experiential avoidance tendencies, reconnect to what they value in life, and set goals to translate these values into committed action, a process known as psychological flexibility [20]. While acceptance may target psychotic distress, components of commitment can potentially improve reward-related motivational deficits, making ACT a promising intervention for early psychosis.

Studies have indicated that ACT is a feasible and accepted approach in both inpatient and outpatient samples with established psychosis [25-28], with promising results of ACT in comparison to treatment as usual on various clinical and functional outcomes [25-27,29-32]. However, meta-analyses report inconsistent effects of ACT for psychosis on symptoms or clinical outcomes [33,34]. The mixed evidence of the current ACT for psychosis literature may stem from methodological challenges, for example, the heterogeneity due to different intervention protocols and outcomes measured [33,34]. However, the mixed evidence could also reflect a gap between the therapy room and the real world, where patients struggle to apply the skills and techniques learned in therapy in their day-to-day lives due to motivational or functional deficits commonly experienced in early psychosis [11].

One way to facilitate the therapy to real-world transfer is to provide ACT within a blended care approach, combining face-to-face therapy sessions with an ecological momentary intervention (EMI) [35,36]. EMIs deliver real-time psychological interventions in daily life using digital technology [37], and as such, they enable patients to access interventions that are tailored to what they need in a given moment and context. More importantly, by providing real-world and real-time psychological interventions, EMIs aim to produce changes in underlying mechanisms that may ultimately lead to sustainable changes under real-world conditions [35]. In psychosis, the use of EMIs to deliver treatment has been shown feasible and acceptable with high compliance and satisfaction rates [38-45], indicating a potential avenue to expand ACT therapy for psychosis beyond traditional therapy settings.

Given the potential of blended care interventions for psychosis, we have developed the ACT in daily life (ACT-DL) intervention, that deploys the use of the ACT-DL EMI in addition to face-to-face sessions with a trained ACT-therapist [46]. The ACT-DL EMI takes the form of a smartphone app that allows patients to practice ACT skills in between therapy sessions, at times when they most need it. Furthermore, the app prompts individuals multiple times a day to fill in short experience sampling method (ESM) [47-49] questionnaires on current affect, context, and behavior with the aim of increasing emotional awareness, followed by an ACT exercise or visual cue of an ACT metaphor. A pilot study has tested ACT-DL and found very good completion rates, use of exercises, and positive user experiences, in a heterogeneous clinical sample of patients with mental disorders [50]. Furthermore, group-based ACT-DL in emerging adults with subclinical symptoms of depression and psychosis was feasible and led to a significant reduction in clinically rated depression relative to an active control condition [51]. In a subsequent multicentered randomized controlled trial known as the INTERACT trial [52], individual ACT-DL was tested in 148 individuals with early psychosis, yielding promising results on efficacy outcomes [53]. However, treatment efficacy must be interpreted alongside treatment engagement and acceptability.

Effective treatment uptake and engagement are important prerequisites for therapy to be successful and may depend on whether the therapy is acceptable to and inclusive of all individuals taking part in the therapy [54-56]. Engaging individuals in treatment for early psychosis has proven challenging, with a meta-analysis reporting disengagement rates between 1% and 41% [57]. Moreover, meta-analyses have identified several predictors of disengagement, such as substance use, poor medication adherence, symptom severity, and minority status, although evidence on employment status, age, and gender is mixed [57]. Understanding treatment engagement in both face-to-face therapy and EMIs is crucial for interpreting treatment outcomes, exploring the role of blended care in treatment engagement, as well as to identifying key predictors to better target individuals who are more likely to disengage, ultimately improving treatment efficacy and delivery.

In this study, we aimed to investigate among participants in the intervention arm: (1) treatment engagement with both the face-to-face sessions and the EMI part of ACT-DL; (2) acceptability of the ACT-DL intervention as a whole and the metaphors and exercises in the app; and (3) whether demographic, clinical, or functional characteristics predict treatment engagement and acceptability. We hypothesize that (1) participants show good engagement with ACT-DL, (2) participants will perceive ACT-DL as acceptable, and (3) different characteristics, such as demographical and clinical characteristics, predict treatment engagement and acceptability. We believe that our results will induce useful intervention optimization recommendations, which may ultimately lead to improved treatment engagement, acceptability, and efficacy, as well as a higher potential for clinical implementation.

## Methods

### Study Design and Participants

In the multicenter INTERACT randomized controlled trial (OMON NL46439.068.13), individuals with UHR or FEP were randomly allocated (1:1) to ACT-DL in addition to treatment-as-usual (TAU) as the experimental condition or a control condition of TAU only, which included routine mental health care. The aim of the INTERACT trial was to test the efficacy of ACT-DL on reducing psychotic distress, psychotic experiences, psychopathology, and social functioning, as well as to evaluate treatment acceptability, adherence, and fidelity [52]. Based on power calculations, we aimed to recruit 150 participants in secondary mental health services in 5 regions in the Netherlands and Flanders (Belgium): Amsterdam, The Hague, Maastricht/Eindhoven, Flemish-Brabant, and East/West-Flanders. Between June 2015 and December 2018, individuals receiving care from these secondary mental health services were informed about the study by their treating clinician, and if interested, were approached by a member of the research team who provided further information. A full eligibility assessment was conducted by the researcher after informed consent was obtained (see Ethical Considerations section). Inclusion criteria were (1) aged 15-65 years, (2) UHR (without prior use of antipsychotic medication for psychotic symptoms) or FEP (onset within last 3 years) as assessed by the Comprehensive Assessment of At Risk Mental States [1] and the Nottingham Onset Schedule [58], (3) good command of the Dutch language, and (4) ability to provide written informed consent. Exclusion criteria were (1) primary diagnosis of alcohol or substance abuse as established with the Mini International Neuropsychiatric Interview [59], and (2) severe endocrine, cardiovascular, or brain disease. This secondary analysis only focuses on individuals randomly allocated to the intervention arm (ACT-DL + TAU) of the INTERACT trial. The study protocol has been published elsewhere [52].

### ACT-DL Intervention

The manualized ACT-DL intervention consisted of 8 ACT training sessions administered face-to-face by a clinician (psychologist) trained in ACT, each for around 45-60 minutes, and an ACT-based EMI, over an 8-week intervention period (Multimedia Appendix 1). The intervention included a session for psychoeducation, followed by 6 ACT sessions aimed to enhance participants’ psychological flexibility by training them in a new ACT component each week (ie, creative hopelessness, acceptance, defusion, self and mindfulness, values, and committed action), which were integrated and reviewed in the last session. The ACT-based EMI was administered through a smartphone-based app (ie, the PsyMate app [60]) to allow participants to apply the skills that they trained in therapy into their daily lives for at least 3 consecutive days per week following (from session 2) each face-to-face session. On each of these days, participants received notifications on the app at 8 semirandom moments, asking them to complete a brief ESM questionnaire on their current mood, psychotic experiences, and activities, with the goal of increasing emotional awareness. Participants were then offered, with a 50:50 ratio, either an ACT exercise or metaphor training them in the ACT component covered in the face-to-face session. After participants were trained in each ACT component separately, the EMI was extended to cover the full range of components in order to train participants to adopt ACT skills and techniques flexibly depending on the context. Participants could initiate an on-demand ACT exercise whenever they were struggling with difficult thoughts or emotions. They were also asked to initiate and complete a morning and evening questionnaire every day. In addition to the app, they could also do ACT exercises in a paper workbook. Participants had no longer access to the ACT-DL EMI after the completion of the intervention period. The ACT-DL intervention procedure is described elsewhere [46].

### Ethical Considerations

This study was conducted in accordance with ethical principles for research involving human participants. The INTERACT study was approved by the Medical Ethics Review Committees at Maastricht University Medical Center in the Netherlands (NL46439.068.13) and the University Clinic Leuven in Belgium (B322201629214). Study participants were informed about the study procedures in person or by phone (including secondary analyses of collected data) and were given time to consider participation. Written informed consent was obtained from each participant prior to assessment and randomization and could be withdrawn by participants at any time. Participants were then deidentified and were allocated a pseudonymized study ID. Safety was monitored throughout the study period, as detailed in the study protocol [52]. Participants were reimbursed according to their attendance at outcome assessment appointments, with increasing amounts for later time points (up to 145 euros in gift vouchers, approximately US $167 based on the exchange rate at the time of the study, in 2020), as well as for additional travel expenses. There was no reimbursement provided for the therapy sessions or the app use during the intervention period.

### Measures

#### Treatment Engagement

We based treatment engagement numbers of the face-to-face sessions on information from treatment integrity questionnaires that were sent to the research department at the end of each treatment combined with email contacts between an independent researcher and the trained clinician. Treatment engagement with the app was based on app use data for each participant, including a completed number of ESM questionnaires, (on-demand) exercises, metaphors, and morning and evening questionnaires. Participants could fill in a maximum of 24 ESM questionnaires per intervention week, followed each by an ACT exercise or visual cue of an ACT metaphor. The number of self-initiated on-demand exercises was unlimited. Morning and evening questionnaires were available each morning and evening between therapy sessions, resulting in a theoretical maximum of 7 questionnaires each to self-initiate between sessions. Therapy sessions were in theory weekly. In reality, in some cases, more than 7 days could pass between two subsequent sessions. In those instances, participants used the app more than a week in between therapy sessions, resulting in a higher number of morning and evening questionnaires.

#### Acceptability

Intervention acceptability was assessed with a bespoke 9-item debriefing questionnaire that participants filled in postintervention. Higher scores indicated higher acceptability, except for 2 items on the burden of the app in terms of both the number and length of items within ESM questionnaires, for which higher scores reflected a higher level of burden. For the exploratory analysis, we created three subscales: acceptability of the (1) ACT training and (2) PsyMate ACT-DL app, and (3) notification burden. Second, momentary acceptability of the app metaphors was assessed with the item “How useful is this metaphor for you right now?” and daily acceptability of the app exercises with the item “How useful were the exercises for you today?” All items were measured on Likert scales with scores ranging 1-7 (1=not at all; 4=average; and 7=very much).

#### Prediction of Treatment Engagement and Acceptability

Demographic characteristics included age, gender (0 male; 1 female), ethnicity (0 nonminority background; 1 minority background), and educational achievement (0 no higher education; 1 higher education). Participants who themselves or whose parents (at least one) were not born in Belgium or the Netherlands were defined as having a minority background. Higher education was defined as having obtained a bachelor’s or master’s degree. Psychotropic medication use (0 no use; 1 use) was assessed with a study-specific questionnaire on current medication use and included among others use of antipsychotics (FEP only), antidepressants, and anxiolytics. Premorbid baseline IQ was assessed with the Dutch Adult Reading Test [61,62], of which age- and gender-corrected IQ scores were used (range: 55-145). Baseline symptom severity was assessed with the Brief Psychiatric Rating Scale (previous 2 weeks) [63] affect (range 5:35), activation (range: 7-49), negative (range: 6-42), and positive symptom (range: 6-42) subscale scores. Baseline functioning was measured with the Dutch version of the Social and Occupational Functioning Scale (previous 2 weeks) [64]. Interrater reliability analysis for the total INTERACT sample demonstrated sufficient agreement with a score of 0.67 for the Social and Occupational Functioning Scale and scores ranging between 0.81 and 0.95 for the Brief Psychiatric Rating Scale subscales [53].

### Statistical Analysis

As for the treatment engagement with the face-to-face sessions, the frequency distribution and the mean number of sessions attended were calculated. As for treatment engagement with the app, the weekly sample mean number of various interactions with the app was calculated. An overall number of ACT-DL app interactions included completed morning, evening, and ESM questionnaires, as well as completed on-demand exercises. Weekly missingness due to therapy dropout (ie, the patient did not show up for the session), therapist-related causes (ie, the therapist forgot to log into the PsyMate or to send the data after the participant used it), or technical issues (eg, the participant did not receive any beeps) was excluded. Missingness due to participant-related causes (ie, the participant was not motivated or too ill to use the app) was recoded as zero. This analysis approach was chosen given that only the latter type of missingness could be assumed to be related to nonengagement with the app.

The sample mean of acceptability scores of the debriefing questionnaire items and of the usefulness scores of the app metaphors (ie, reported after a notification) and exercises (ie, reported in the evening) was calculated. Likert plots were designed to inspect the frequency distribution of acceptability scores of these same elements.

Multiple linear regression models were fitted to investigate whether premorbid IQ, as well as various sociodemographic (ie, age, gender, ethnicity, minority background, and educational achievement), clinical (ie, symptom severity, UHR or FEP status, and psychotropic medication use) and functional characteristics, predicted the number of sessions attended, the weekly mean number of completed ESM questionnaires, completed on-demand exercises, the scores on the debriefing questionnaire subscales, and finally the person-mean app metaphor and exercise usefulness scores. All statistical analyses were conducted in Stata (version 14; StataCorp) [65].

## Results

### Sample Characteristics

Of the 148 participants in the total INTERACT trial, 71 (48%) participants were randomized in the experimental condition (ACT-DL+TAU). Participants had a mean age of 26 (SD 6) years, with slightly more women (n=42, 59%) than men in the sample (Table 1).

**Table 1:** Baseline sample characteristics (n=71).

Measure			Participants
**Demographics**			
	**Age (years)**		
		Mean (SD)	26 (6)
		Range	16-47
	Sex (female), n (%)		42 (59)
	Education (high), n (%)		27 (38)
	Minority background, n (%)		26 (37)
**Clinical characteristics**			
	Early psychosis status (FEP^a^), n (%)		36 (51)
	Psychotropic medication, n (%)		44 (62)
	**DART** ^b^ **IQ**		
		Mean (SD)	95.10 (12.34)
		Range	61-127
	**BPRS** ^c^ **(positive symptoms)**		
		Mean (SD)	9.34 (3.32)
		Range	6-18
	**BPRS (negative symptoms)**		
		Mean (SD)	8.45 (2.68)
		Range	6-17
	**BPRS (affective symptoms)**		
		Mean (SD)	12.55 (4.44)
		Range	5-23
	**BPRS (activation symptoms)**		
		Mean (SD)	9.13 (2.19)
		Range	7-17
	**SOFAS** ^d^		
		Mean (SD)	43.92 (10.35)
		Range	21-80

^a^FEP: first episode of psychosis.

^b^DART: Dutch Adult Reading Test.

^c^BPRS: Brief Psychiatric Rating Scale.

^d^SOFAS: Social and Occupational Functioning Scale.

### Treatment Engagement

As to treatment engagement (Figure S1 in Multimedia Appendix 2) with the sessions, 42 of 71 (59%) participants completed all 8 face-to-face ACT-DL sessions, including one psychoeducation session (Tables 2 and 3). In contrast, of the 71 participants, 9 (13%) participants did not attend any face-to-face therapy session, with another 20 (28%) participants dropping out after 1 to 7 sessions. On average, participants completed 6 (SD 3) of 8 sessions. App engagement data were available for 58 participants, with all data missing for 13 participants: 12 because of dropout before the start of the first ACT session (ie, session 2) and one because of technical issues. Data for all 7 ACT-DL app study weeks were available for 18 participants, with data missing for some weeks for 40 participants due to various reasons. On a weekly basis, participants had on average 13 (SD 8.7) ACT-DL app interactions, including 6 of 24 (SD 5.0) ESM questionnaires (8 notifications per day the first 3 days after therapy), indicating response compliance to the notifications of 25% (Figure S2 in Multimedia Appendix 2).

**Table 2:** Treatment engagement based on the completion of the face-to-face sessions.

Sessions	Value (n=71), n (%)
0	9 (13)
1	3 (4)
2	3 (4)
3	4 (6)
4	3 (4)
5	1 (1)
6	1 (1)
7	5 (7)
8	42 (59)

**Table 3:** Treatment engagement based on completion of the app^a^.

Interactions		Mean (SD)	Range
Total		12.7 (8.7)	0-38
**Programmed**			
	Morning questionnaires	2.1 (2.0)	0-8
	Evening questionnaires	2.0 (1.9)	0-8
	ESM^b^ questionnaires	5.6 (5.0)	0-16
	Exercises	2.9 (2.6)	0-8
	Metaphors	2.4 (2.3)	0-7
**On-demand** ^c^			
	Exercises “yes”	3.0 (3.3)	0-14
	Exercises “no, later”	1.3 (1.3)	0-7

^a^There were n=3 participants who did not have any data due to a lack of motivation to use the app.

^b^ESM: experience sampling method.

^c^Participants initiating an on-demand exercise were asked “Do you want to do an ACT exercise right now?” upon which they then either clicked “Yes” or “No, later.”

### Acceptability

**Figure 1:**
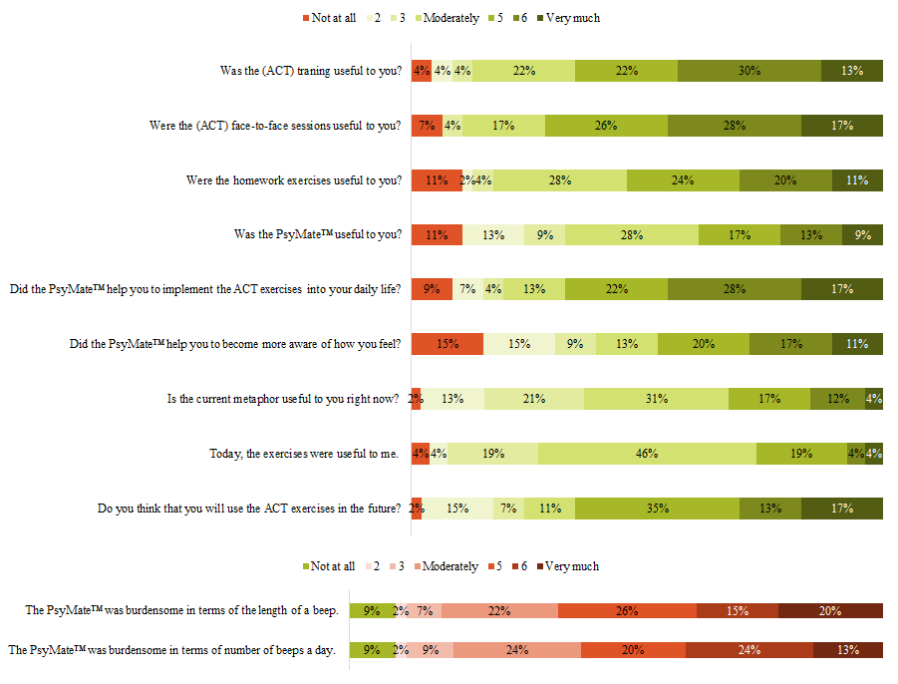
Acceptability and burden of the ACT-DL intervention. ACT: acceptance and commitment therapy; ACT-DL: acceptance and commitment therapy in daily life.

From the 46 participants who completed the debriefing questionnaire (Figure 1), almost all indicated that the training in general (n=44, 96%; mean 4.96, SD 1.53), as well as the face-to-face ACT sessions (n=43, 93%; mean 5.11, SD 1.55) and the homework exercises (n=41, 89%; mean 4.54, SD 1.70) were to some extent useful (score≥2). Furthermore, almost all participants felt that the ACT-based EMI in general had been useful (n=41, 89%; mean 4.02, SD 1.77) to some extent (score≥2) and helped to apply the ACT exercises in their daily lives (n=42, 91%; mean 4.87, SD 1.82) and to increase emotional awareness (n= 39, 85%; mean 4.02, SD 2.01). Almost all participants indicated that both the number of notifications a day (n=42, 91%; mean 4.78, SD 1.74) and the number of items within a notification (n=42, 91%; mean 4.67, SD 1.70) were to some extent (score≥2) burdensome. Among the participants, 9% (n=4) said not to be burdened at all (score 1).

As for the ACT exercises, almost all participants acknowledged having used the ACT-based EMI (n=41, 89%) or the workbook (n=39, 85%) at least once, and 48% (n=22) reported having done at least one exercise autonomously (ie, without the app or workbook). From the participants, 98% (n=45) of participants indicated that they at least to some extent (score≥2) intended to use these exercises in the future (mean 4.70, SD 1.70). While the majority of individuals used either all 3 methods (n=18, 39%; Figure S3 in Multimedia Appendix 2) or both the app and the workbook (n=18, 39%) to practice the exercises, a smaller percentage of individuals used either the app alone (n=5, 11%) or the workbook in combination with autonomous training (n=3, 7%). Only 1 (2%) participant reported to have performed the exercises autonomously, and another participant did not indicate any method.

When turning to findings on the acceptability of the ACT-based EMI (n=58), 98% (n=57) of the participants indicated that the metaphors (mean 3.96, SD 1.34) were at least to some extent useful to them (score≥2). As for the average usefulness of the exercises, 96% (n=56) of the participants rated the exercises (mean 4.05, SD 1.13) as useful to them to at least some extent (score≥2).

### Predictors of Treatment Engagement and Acceptability

In the multiple linear regression models (Table 4 and Multimedia Appendix 3) with treatment engagement as outcome variables, ethnic minority background significantly predicted lower response compliance to the ESM questionnaire notifications (B=–4.37; *P*=.01).

**Table 4:** Predicting treatment engagement with the ACT-DL^a^ sessions, notifications, and on-demand exercises based on demographic and clinical characteristics.

	Sessions^b^ (n=69)			Notifications^c^ (n=56)				On-demand^d^ (n=56)		
	B^e^ (SE)	β^f^	*P* value	B (SE)	β	*P* value	B (SE)		β	*P* value
FEP^g^	–0.74 (1.40)	–0.12	.60	–2.69 (2.27)	–0.27	.24	1.13 (1.57)		0.17	.48
Age	–0.06 (0.07)	–0.11	.44	0.22 (0.14)	0.22	.12	–0.03 (0.10)		–0.05	.75
Female	0.95 (0.84)	0.15	.26	–0.37 (1.48)	–0.03	.80	1.08 (1.02)		0.15	.30
Minority	–0.14 (0.93)	–0.02	.88	–4.37 (1.66)	–0.40	.01	–0.11 (1.15)		–0.02	.92
Education	1.47 (1.05)	0.23	.17	–2.17 (1.77)	–0.21	.23	–0.94 (1.22)		–0.14	.45
Psychotropic medication	0.80 (1.22)	0.13	.51	1.45 (1.95)	0.13	.46	–0.61 (1.35)		–0.09	.65
DART^h^	–0.01 (0.04)	–0.05	.75	–0.05 (0.07)	–0.12	.46	0.07 (0.05)		0.26	.15
BPRS^i^ positive	0.08 (0.14)	0.09	.54	0.32 (0.23)	0.22	.16	–0.07 (0.16)		–0.07	.65
BPRS negative	0.01 (0.16)	0.00	.98	0.10 (0.29)	0.05	.73	–0.25 (0.20)		–0.20	.22
BPRS affective	–0.01 (0.11)	–0.01	.95	–0.16 (0.19)	–0.15	.41	–0.06 (0.13)		–0.09	.63
BPRS activation	–0.25 (0.20)	–0.18	.20	0.20 (0.32)	0.09	.54	–0.10 (0.22)		–0.07	.64
SOFAS^j^	0.03 (0.05)	0.10	.51	0.08 (0.07)	0.18	.27	–0.06 (0.05)		–0.20	.25

^a^ACT-DL: acceptance and commitment therapy in daily life.

^b^Number of sessions attended.

^c^Number of ESM notifications filled in.

^d^Number of on-demand exercises to which individuals said yes.

^e^B: unstandardized coefficients.

^f^β: standardized coefficients.

^g^FEP: first episode of psychosis.

^h^DART: Dutch Adult Reading Test [61,62].

^i^BPRS: Brief Psychiatric Rating Scale [63].

^j^SOFAS: Social and Occupational Functioning Assessment Scale [64].

In contrast, in the models with treatment acceptability outcome variables (Table 5), ethnic minority backgrounds predicted higher perceived app usefulness (B=1.25; *P*=.049). Moreover, being female predicted a lower perceived in-the-moment usefulness of the visual cues of the metaphors (B=–1.14; *P*=.005). While affective symptom severity predicted higher perceived usefulness of the ACT training (B=0.14; *P*=.04), negative symptom severity predicted lower perceived usefulness of this subscale (B=–0.26; *P*=.01). No other predictors reached significance.

**Table 5:** Predicting acceptability of the ACT^a^ training and the PsyMate app based on demographic and clinical characteristics.

	ACT training^b^ (n=44)				PsyMate general^c^ (n=44)				PsyMate burden^d^ (n=44)				App metaphors^e^ (n=50)				App excercises^f^ (n=50)		
	B^g^ (SE)	β^h^	*P* value	B (SE)		*β*	*P* value	B (SE)		*β*	*P* value	B (SE)		*β*	*P* value	B (SE)		*β*	*P* value
FEP^i^	1.50 (0.81)	0.50	.08	0.09 (0.85)		0.03	.92	1.03 (0.76)		0.36	.18	0.20 (0.61)		0.07	.75	0.56 (0.52)		0.24	.29
Age	0.03 (0.04)	0.10	.53	0.03 (0.05)		0.10	.54	–0.04 (0.04)		–0.16	.32	0.02 (0.03)		0.07	.62	0.04 (0.03)		0.16	.29
Female	0.08 (0.49)	0.03	.87	0.37 (0.51)		0.11	.48	0.79 (0.46)		0.25	.10	–1.14 (0.38)		–0.40	.005	–0.34 (0.34)		–0.14	.32
Minority	0.53 (0.59)	0.16	.38	1.25 (0.61)		0.36	.049	0.53 (0.55)		0.17	.34	0.60 (0.45)		0.20	.19	–0.07 (0.39)		–0.03	.86
Education	–0.67 (0.63)	–0.22	.30	–0.20 (0.65)		–0.06	.76	0.27 (0.58)		0.09	.65	–0.63 (0.48)		–0.23	.20	–0.46 (0.40)		–0.20	.27
Psychotropic medication	–1.03 (0.67)	–0.32	.14	–0.67 (0.70)		–0.20	.34	–0.70 (0.62)		–0.23	.27	–0.49 (0.51)		–0.17	.35	–0.41 (0.45)		–0.17	.37
DART^j^	–0.02 (0.02)	–0.18	.34	–0.02 (0.02)		–0.17	.35	0.02 (0.02)		0.13	.48	0.02 (0.02)		0.16	.32	–0.01 (0.02)		–0.11	.50
BPRS^k^ positive	0.00 (0.08)	–0.01	.95	0.01 (0.08)		0.02	.93	–0.04 (0.07)		–0.11	.54	–0.03 (0.06)		–0.08	.59	–0.02 (0.05)		–0.05	.77
BPRS negative	–0.26 (0.10)	–0.49	.01	–0.19 (0.10)		–0.32	.07	–0.05 (0.09)		–0.10	.56	–0.01 (0.08)		–0.02	.89	0.04 (0.07)		0.09	.60
BPRS affective	0.14 (0.07)	0.44	.04	0.02 (0.07)		0.05	.80	0.12 (0.06)		0.39	.06	–0.09 (0.05)		–0.31	.08	–0.09 (0.04)		–0.35	.06
BPRS activation	–0.10 (0.11)	–0.15	.36	–0.06 (0.12)		–0.09	.60	–0.13 (0.10)		–0.19	.24	–0.06 (0.08)		–0.11	.44	0.02 (0.07)		0.04	.80
SOFAS^l^	0.02 (0.03)	0.16	.42	0.00 (0.03)		–0.01	.94	0.04 (0.03)		0.26	.16	0.03 (0.02)		0.22	.17	0.00 (0.02)		0.01	.94

^a^ACT: acceptance and commitment therapy.

^b^Items: Was the training useful to you? Were the face-to-face sessions useful to you? Were the homework exercises useful to you?

^c^Items: Was the app useful to you? Did the app help you to implement the ACT exercises into your daily life? Did the app help you to become more aware of how you feel?

^d^Items: the app was burdensome in terms of the length of a beep. The app was burdensome in terms of number of beeps a day.

^e^(on the app) How useful is this metaphor for you right now?

^f^(on the app) How useful were the exercises today?

^g^B: unstandardized coefficients.

^h^β: standardized coefficients.

^i^FEP: first episode of psychosis.

^j^DART: Dutch Adult Reading Test [61,62].

^k^BPRS: Brief Psychiatric Rating Scale [63].

^l^SOFAS: Social and Occupational Functioning Assessment Scale [64].

## Discussion

### Principal Findings

This is the first study to investigate treatment engagement with and acceptability of ACT-DL in an early psychosis sample. We found good treatment engagement when taking into account the attendance to face-to-face sessions and weekly interactions with the app in absolute numbers, yet the proportion of individuals who attended all face-to-face sessions, as well as the proportion of completed ESM questionnaires was low in comparison to reports from previous studies. Our acceptability data showed a positive view of all elements of the intervention including the face-to-face sessions and the ACT-based EMI, suggesting that ACT-DL helped participants apply ACT exercises and increase emotional awareness in daily life, despite a perceived burden by number and length of notifications. Individual differences in demographic and baseline clinical characteristics predicted treatment engagement and acceptability. That is, while ethnic minority status predicted lower notification response compliance, it predicted higher app acceptability. Furthermore, being female predicted lower perceived app metaphor usefulness. Negative symptoms predicted lower ACT training acceptability, while we found the opposite for affective symptoms.

### Treatment Engagement and Acceptability

The proportion of individuals completing all face-to-face sessions is similar to that found in a previous CBT [66] for psychosis study where treatment disengagement was also defined as no show before or during therapy. However, it is lower than that found in other studies investigating the efficacy of (blended) ACT for psychosis [25-27,31], in a meta-analysis of psychosocial interventions [67] for psychosis, and in a blended care intervention in a psychosis sample [45], where completion rates ranged between 76% and 100%. It is of note that only one of those studies [27] had a comparable treatment format with 8 individual ACT sessions offered. In contrast, the other studies mentioned did include individuals at the later stages of psychosis, had a smaller sample size, and offered only up to 4 sessions [25,26,31,45], limiting the comparability of these findings. Nevertheless, one potential reason for the discrepancy in completion rates may be that we included individuals in the early stages of psychosis, and previous studies have shown high psychotherapy dropout in FEP [66,68-70] individuals and high service disengagement in UHR individuals [71-73].

Second, the ACT-DL intervention manual and EMI were standardized and generic and did not allow for much flexibility and personalization of the treatment, which may have hampered adequate treatment engagement for some individuals. In this respect, a recent meta-analysis showed that in comparison to generic CBT for psychosis, personalized and targeted CBT for psychosis was more effective in alleviating distress related to auditory hallucinations [74]. In that same vein, a recent pilot trial [45] used ESM questionnaire data as input for subsequent functional analysis of voice-hearing and voice-related coping in the face-to-face sessions and used the EMI to provide personalized coping reminders in the daily lives of participants, with excellent treatment engagement and acceptability. As such, a fruitful alteration to the ACT-DL protocol used in this study could be to shift toward a more personalized and formulation-based intervention approach where a functional diagnostic analysis of the patient’s difficulties and not manually steer the content and the order of the sessions and the app. At the same time, the potential of the EMI may further improve by advanced personalization of the ACT-DL app itself, which we will hint at below.

Treatment engagement with the ACT-DL app may be approached from two different angles. That is, when looking at the level of engagement with the ACT-DL app as the number of ACT exercises or metaphors performed or viewed, our results suggest that the app helped participants to engage in their therapeutic trajectory on average 9 times a week (6 exercises or metaphors followed after a prompt in addition to 3 on-demand exercises). This number is comparable to other ACT-based EMI [50,51,75] or EMI for psychosis studies [41], where participants performed on average 4 to 12 prompted or self-initiated exercises a week. However, do note that direct comparisons with other EMIs for psychosis are difficult to make due to different definitions of response compliance [42,44]. In contrast, the mean response compliance to the ESM questionnaires was clearly lower than that found in other blended interventions where monitoring of affect, symptoms, and their context was (part of) the intervention and where the number of prompts was comparable [45,51,76]. The lower response compliance could reflect the perceived burden of the notification schedule, as was also indicated in the debriefing questionnaire. Notification burden may be attributable to various reasons: the notification schedule nor the content of the items was personalized, and there was no feedback provided after ESM monitoring. In this respect, the optimal number of notifications may not be the same for every patient and a person-tailored beep schedule may prove an effective addition to our intervention. An additional alteration would be to improve the therapy to real-world transfer by providing personalized feedback to patients based on the questionnaire data on affect, context, activities, and ACT skills, which may then function as a starting point for the next ACT face-to-face session. These alterations could potentially increase response compliance to the ESM questionnaires, and more broadly, treatment engagement with the ACT-DL app in general.

Despite the perceived notification burden, our results indicate the acceptability of the ACT-DL intervention with positive views on all of its elements, including the face-to-face sessions, which is in line with previous findings on studies investigating the acceptability of ACT [25-27], EMIs [41,42,76-78], blended care [45] for psychosis, and ACT in a blended care format [46,50,79]. Our results showed that the app helped participants to apply the ACT exercises and to increase emotional awareness in their daily lives, suggesting that ACT-DL does indeed improve the therapy to real-world transfer. Second, almost all individuals indicated to have used the app in combination with the workbook to do the exercises, encouraging the continued use of a blended care approach in early psychosis, instead of offering the intervention as a stand-alone EMI.

### Predicting Treatment Engagement and Acceptability

Acceptability scores showed substantial variability, which was partly predicted by negative and affective symptom severity, ethnic minority (higher app usefulness), and gender (being female predicted lower usefulness of the visual metaphor). Negative symptoms predicted lower training usefulness, whereas affective symptom severity predicted higher training usefulness.

In this study, ethnic minority status was a significant predictor of lower compliance to the ACT-based EMI and negative symptom severity predicted lower acceptability of the ACT training. These results are in line with a recent review that linked negative symptom severity with decreased treatment engagement with digital interventions [80], and with other studies showing that ethnic minority status and negative symptom severity are predictors of higher dropout and may hamper treatment effects in regular psychotherapies, as well as in EMIs for psychosis [44,68,81-87]. These results align with a study that assessed engagement with a CBT-based intervention for psychosis, the Actissist app, that found that White ethnicity was associated with higher levels of engagement [88]. Interestingly though, we found that ethnic minority background was related to higher perceived usefulness of the ACT-DL app. One explanation here could be that there was fewer acceptability data available than there was for treatment engagement with the app, potentially inflating acceptability results. Another explanation could be that individuals with a minority background, who may experience more stigma around mental health disorders [89], view the app as a low-barrier form of care, indicating that engagement with and acceptability of the treatment may not necessarily be related and should be considered as two independent factors in intervention evaluation and optimization.

In any case, understanding how we can culturally adapt our intervention in dialogue with ethnic minority individuals is especially important in early psychosis, given that having an ethnic minority background is a known risk factor for the disorder [90]. In this respect, one study looked into specific challenges to delivering ACT to consumers of mental health services from underserved and underrepresented backgrounds and provided recommendations on how to address these challenges [91]. At the same time, it has been shown that cultural adaptation of interventions is feasible and that it can enhance patient engagement and outcomes [92]. As such, tailoring the intervention to individuals of various backgrounds will be an important addition to increasing the inclusiveness of our intervention.

Furthermore, the intervention may have been more demanding for participants with more negative symptoms. The intervention format was not specifically adapted for psychosis nor negative symptoms in psychosis specifically. It is thus possible that individuals with more pronounced negative symptoms need more sessions, and that their therapy should focus primarily on the commitment skills within ACT. Although speculative, these adaptations may improve acceptability for individuals with more negative symptoms in particular. A recent meta-analysis revealed varying effects of negative symptoms, indicating that both lower (no perceived need) and higher levels of symptoms are associated with disengagement [57]. These findings may suggest a window of opportunity for effectively targeting these individuals. Therefore, personalization and tailoring of intervention components, such as questionnaire items, exercises, and metaphors to patients’ personal needs, preferences, and symptoms, could enhance the impact of these interventions. Further understanding of characteristics that affect treatment engagement, as well as acceptability, is crucial to optimize and personalize current interventions, which in turn, may improve treatment efficacy.

### Limitations

Some limitations need to be considered. First, we did not assess motivation, nor satisfaction (eg, with the System Usability Scale [93]) with technology (ie, the smartphone app). As to the first, it is possible that some individuals are more open than others to adopt new technologies, which may be an important predictor of treatment engagement in itself [56,94]. As to the latter, this information could have informed us better about the user experience of our app. Second, it is also possible that, due to a lack of debriefing questionnaire data for 25 participants who dropped out before postintervention, acceptability ratings were inflated. Third, we had missing user data due to technical or practical difficulties during the intervention. Future studies investigating blended care interventions should closely monitor the functionality, stability, and fidelity of the intervention while providing extensive training on its use to therapists [95]. Finally, we did not include participants from the control condition, so we cannot conclude whether engagement with the face-to-face sessions was greater in the blended care format.

### Conclusions

The ACT-DL intervention showed promise despite low compliance to the ESM beeps, with participants attending an encouraging number of face-to-face sessions and weekly interactions, suggesting effective real-world application of ACT techniques and improved emotional awareness. These findings support the value of a blended approach for early psychosis and highlight the importance of personalizing interventions based on symptom severity and demographic factors. Future efforts should focus on collaboration with individuals with lived experiences to refine and optimize the intervention for better clinical implementation.

## Appendix

Multimedia Appendix 1 ACT-DL (acceptance and commitment therapy in daily life) intervention components.

Multimedia Appendix 2 Engagement with ACT-DL.

Multimedia Appendix 3 Zero-order correlations and p-values between treatment engagement and acceptability outcomes and predictors of interest.

## Abbreviations

ACT: acceptance and commitment therapy
ACT-DL: acceptance and commitment therapy in daily life
CBT: cognitive behavioral therapy
EMI: ecological momentary intervention
ESM: experience sampling method
FEP: first episode of psychosis
TAU: treatment as usual
UHR: ultrahigh risk

## Data Availability

The dataset generated during this study are available from the corresponding authors upon a reasonable request through the Data curation for OPen Science (DROPS) system using the REDCap (Research Electronic Data Capture) electronic data capture tool [96,97], hosted by the Center for Contextual Psychiatry, KU Leuven. Interested researchers can submit an abstract, which will be reviewed by the research team to avoid overlap with ongoing research projects. After approval, a variable access request is made, and researchers are required to preregister their analysis plan. A data manager will then provide the dataset including the variables for the proposed analysis, accompanied by a time-and date-stamped receipt of data access.
