# Supplementary material for: Engagement and Acceptability of Acceptance and Commitment Therapy in Daily Life in Early Psychosis: Secondary Findings From a Multicenter Randomized Controlled Trial
Source: JMIR Form Res. 2024 Nov 21;8:e57109. doi: 10.2196/57109 (PMC11621719; doi:10.2196/57109)
Supplement: Multimedia Appendix 1 [file formative_v8i1e57109_app1.docx]

The ACT-DL intervention consisted of eight face-to-face ACT sessions combined with the use of an ACT-based EMI. The intervention started with a session for psychoeducation on the Ultra High Risk or First Episode of Psychosis state. Each of the following face-to-face session, led by a trained ACT clinician, introduced a new ACT component or skill, in a predefined sequence: creative hopelessness, acceptance, defusion, self as context, values, committed action. All ACT skills were integrated and discussed in a final session. To further encourage practicing these ACT-skills between sessions, participants used the PsyMate app, a dedicated device that prompts participants to engage with the ACT-DL intervention through questionnaires, exercises and metaphors.

In the first three days after each session, participants received beep-questionnaires at eight random times throughout the day to report mood, symptoms and activities, aiming to enhance mindfulness. In addition, participants received morning and evening questionnaires to further reflect on sleep quality and to evaluate the day. Each beep-questionnaire was followed by (50:50) either a mindfulness exercise they learned in one of the previous sessions, or by an ACT-metaphor, both of which focused on acceptance and defusion skills. In the sixth week, participants explored personal values during their face-to-face session and identified key values. For three consecutive mornings, the app instructed them to select a personal value, set feasible goals related to that value, and commit to them throughout the day, with an evaluation at the day’s end. Additionally, exercises covering all previously discussed components or skills were available on demand in the app for participants to access when needed. The components of the intervention are discussed in greater detail elsewhere [1].

Table S1. Overview of the intervention weeks and components

|  | ACT-DL intervention period | | | | | | | |
| --- | --- | --- | --- | --- | --- | --- | --- | --- |
| Weeks | 1 | 2 | 3 | 4 | 5 | 6 | 7 | 8 |
| ACT sessions | Psychoeducation on Ultra High Risk and First Episode Psychosis | Creative hopelessness | Acceptance | Defusion | Self as context, mindfulness | Values | Committed action | Integration of all skills |
| Beep exercises |  | Mindfulness | Mindfulness, Acceptance | Mindfulness, Defusion | Mindfulness, Self | Mindfulness, Values | Mindfulness, Committed action | All |
| Personal values |  |  |  |  |  | Values | Commitment | Commitment |
| Action exercises |  | Mindfulness, hopelessness | Mindfulness, Acceptance | Mindfulness, Acceptance, Defusion | Mindfulness, Acceptance, Defusion, Self as context | Mindfulness, Acceptance, Defusion, Self as context, Values | All | All |

**References**

1. Vaessen T, Steinhart H, Batink T, et al. ACT in daily life in early psychosis: An ecological momentary intervention approach. *Psychosis*. 2019;11(2):93-104. doi:10.1080/17522439.2019.1578401
