# Supplementary material for: Engagement and Acceptability of Acceptance and Commitment Therapy in Daily Life in Early Psychosis: Secondary Findings From a Multicenter Randomized Controlled Trial
Source: JMIR Form Res. 2024 Nov 21;8:e57109. doi: 10.2196/57109 (PMC11621719; doi:10.2196/57109)

Multimedia Appendix 2: Engagement with ACT-DL

Figure S1. Causes of missing data for 131 study weeks in n=40 participants.


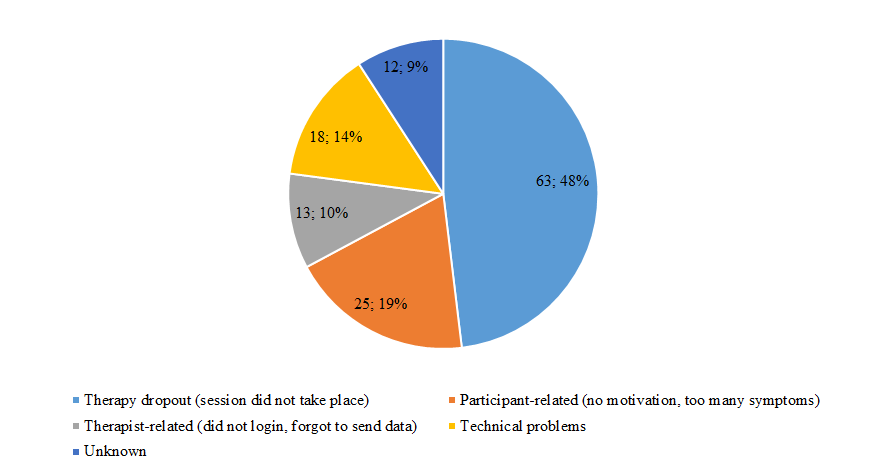


Figure S2. Mean number of interactions per ACT-DL study week, with number of participants for whom data was available.


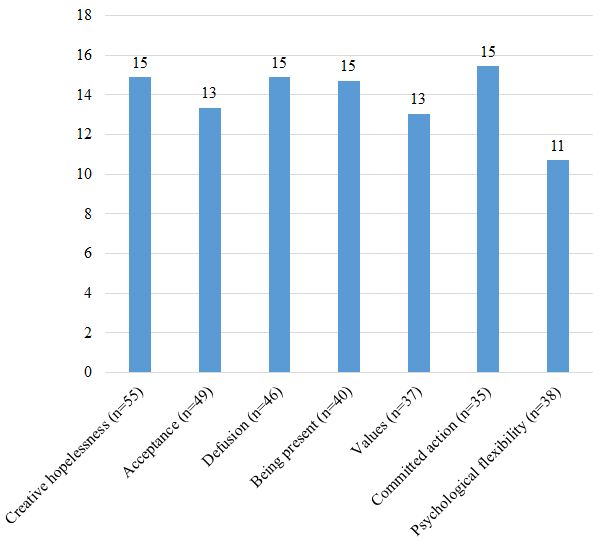


Figure S3. Methods of ACT-DL exercise performance.


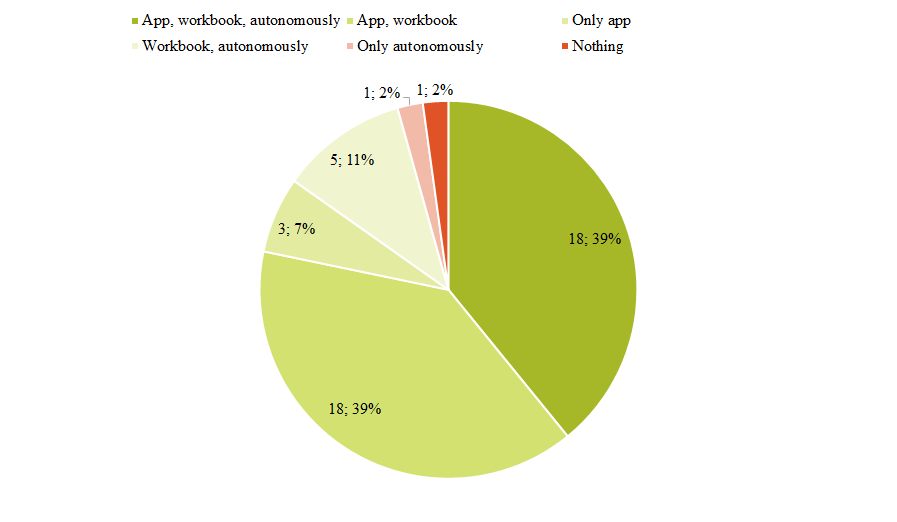

Supplement: Multimedia Appendix 2 [file formative_v8i1e57109_app2.docx]
