# Supplementary material for: Engagement and Acceptability of Acceptance and Commitment Therapy in Daily Life in Early Psychosis: Secondary Findings From a Multicenter Randomized Controlled Trial
Source: JMIR Form Res. 2024 Nov 21;8:e57109. doi: 10.2196/57109 (PMC11621719; doi:10.2196/57109)
Supplement: Multimedia Appendix 3 [file formative_v8i1e57109_app3.docx]

|  | Treatment engagement | | | Treatment acceptability | | | | |
| --- | --- | --- | --- | --- | --- | --- | --- | --- |
|  | Sessions | Notifications | On-demand | ACT training | PsyMate general | PsyMate burden | App metaphors | App exercises |
| FEP | 0.01; *P=*.97 | -0.17; *P=*.20 | 0.15; *P=*.26 | 0.03; *P=*.83 | -0.09; *P=*.55 | -0.01; *P=*.92 | 0.03; *P=*.83 | 0.28; *P=*.04 |
| Age | -0.05; *P=*.70 | 0.06; *P=*.63 | 0.04; *P=*.78 | 0.13; *P=*.39 | 0.05; *P=*.72 | -0.05; *P=*.72 | 0.01; *P=*.92 | 0.09; *P=*.51 |
| Female | 0.13; *P=*.29 | -0.05; *P=*.72 | 0.17; *P=*.21 | 0.05; *P=*.75 | 0.14; *P=*.35 | 0.23; *P=*.12 | -0.39; *P=*.004 | -0.22; *P=*.11 |
| Minority | -0.07; *P=*.58 | -0.29; *P=*.02 | 0.05; *P=*.71 | 0.14; *P=*.36 | 0.41; *P=*.005 | -0.09; *P=*.57 | 0.23; *P=*.10 | 0.16; *P=*.26 |
| Education | 0.20; *P=*.09 | -0.10; *P=*.45 | 0.02; *P=*.88 | -0.10; *P=*.49 | -0.20; *P=*.18 | 0.20; *P=*.18 | -0.16; *P=*.25 | -0.25; *P=*.07 |
| Psychotr. | 0.07; *P=*.54 | -0.02; *P=*.87 | 0.01; *P=*.92 | -0.07; *P=*.62 | -0.14; *P=*.36 | -0.04; *P=*.77 | -0.15; *P=*.30 | 0.05; *P=*.74 |
| DART | 0.10; *P=*.39 | -0.08; *P=*.56 | 0.11; *P=*.42 | -0.12; *P=*.43 | -0.27; *P=*.08 | 0.29; *P=*.06 | -0.03; *P=*.83 | -0.31; *P=*.02 |
| BPRS pos. | 0.02; *P=*.87 | 0.08; *P=*.56 | -0.04; *P=*.75 | 0.00; *P=*.98 | 0.02; *P=*.90 | -0.08; *P=*.61 | -0.17; *P=*.23 | -0.11; *P=*.45 |
| BPRS neg. | -0.03; *P=*.79 | 0.08; *P=*.55 | -0.21; *P=*.11 | -0.28; *P=*.06 | -0.30; *P=*.04 | -0.01; *P=*.93 | -0.08; *P=*.57 | 0.11; *P=*.44 |
| BPRS aff. | 0.04; *P=*.72 | 0.09; *P=*.49 | -0.17; *P=*.20 | 0.03; *P=*.83 | -0.22; *P=*.14 | 0.27; *P=*.07 | -0.34; *P=*.013 | -0.47; *P*<.001 |
| BPRS act. | -0.13; *P=*.29 | 0.14; *P=*.28 | -0.12; *P=*.39 | -0.13; *P=*.38 | -0.03; *P=*.84 | -0.17; *P=*.25 | -0.13; *P=*.38 | -0.06; *P=*.66 |
| SOFAS | 0.14; *P=*.26 | 0.22; *P=*.10 | -0.20; *P=*.14 | 0.02; *P=*.90 | -0.12; *P=*.42 | 0.30; *P=*.04 | 0.06; *P=*.69 | -0.22; *P=*.11 |

DART = Dutch Adult Reading Test [1,2]; BPRS = Brief Psychiatric Rating Scale [3]; SOFAS = Social and Occupational Functioning Assessment Scale [4]; Psychotr. = psychotropic medication; pos. = positive; neg. = negative; aff. = affective; act. = activation; ^a^Number of sessions attended; ^b^Number of ESM notifications filled in; ^c^Number of on-demand exercises to which individuals said yes.

^d^Items: Was the training useful to you? Were the face-to-face sessions useful to you? Were the homework exercises useful to you? ^e^Items: Was the app useful to you? Did the app help you to implement the ACT exercises into your daily life? Did the app help you to become more aware of how you feel? ^f^Items: The app was burdensome in terms of the length of a beep. The app was burdensome in terms of number of beeps a day ^g^(on the app) How useful is this metaphor for you right now? ^h^(on the app) How useful were the exercises today?

References

1. Schmand B, Bakker D, Saan R, Louman J. [The Dutch reading test for adults: A measure of premorbid intelligence level]. *Tijdschr Gerontol Geriatr*. 1991;22(1):15-19.

2. Nelson HE. *National Adult Reading Test (NART) for the Assessment of Premorbid Intelligence in Patients with Dementia: Test Manual*.; 1982.

3. Ventura J, Lukoff D, Nuechterlein KH. Brief Psychiatric Rating Scale (BPRS) Expanded Version (4.0): scales, anchor points and administration manual. *Int J Methods Psychiatr Res*. 1993;3:227-244.

4. Goldman HH, Skodol AE, Lave TR. Revising axis V for DSM-IV: A review of measures of social functioning. *Am J Psychiatry*. 1992;149(9):1148-1156. doi:10.1176/ajp.149.9.1148
